# Do micro-breaks between study sessions enhance Chinese university students’ learning concentration?

**DOI:** 10.3389/fpsyg.2026.1714389

**Published:** 2026-02-10

**Authors:** Haiming Zhou, Laitan Fang, Xinping Song, Wanjuan Yin, Jianli Kang, Yuanyuan Huang, Jie Huang

**Affiliations:** 1 Public Course Teaching Department, Shandong University of Science and Technology, Qingdao, China; 2 Founder Technology College, Peking University, Beijing, China; 3 School of Economic and Management, University of Chinese Academy of Sciences, Beijing, China; 4 Capital United Think Tank Federation, Beijing, China; 5 ZR Holdings Limited, Beijing, China; 6 School of Basic Medical Sciences, Hangzhou Normal University, Hangzhou, China; 7 School of Film and Television, Communication University of Tianjin, Tianjin, China; 8 School of Distance Education, Communication University of China, Beijing, China; 9 Xiangan Special School, Xiamen, China; 10 College of Physical Education, Quanzhou Normal University, Quanzhou, China

**Keywords:** affective events theory, chain mediating model, effort-recovery model, learning concentration, micro-breaks between study sessions, positive emotions, study detachment

## Abstract

Amidst technological advancements in artificial intelligence, rapid economic growth, and intense social transformation, college students are facing unprecedented challenges to their learning concentration. In the field of education, confronted with an era of scarce concentration resources, based on affective events theory and the effort-recovery model, this paper seeks to probe into the positive effects of MBSS on learning concentration, investigate the underlying mechanisms and pathways through which these positive effects occur, and explore factors that may enhance such beneficial outcomes. Using cluster random sampling, a questionnaire survey was conducted among 578 college students from three universities in Shandong province. The results indicate a significant positive correlation between MBSS and learning concentration (*β* = 0.292, *p* < 0.01). This relationship is mediated separately by study detachment and positive emotions (mediation effect values: 0.044, 95% CI = [0.008, 0.105] and 0.059, 95% CI = [0.018, 0.118], respectively), and further explained through a chain mediating model (mediation effect value: 0.013, 95% CI = [0.004, 0.031]). This study holds important theoretical and practical implications for elucidating the positive influence and mechanisms of MBSS on learning concentration, as well as for informing policy-makers and educators in developing relevant strategies and interventions.

## Introduction

The rapid economic growth and societal transformation have intensified educational competition, placing unprecedented pressure on students to thrive in the “Neijuan” learning culture (Wang S. et al., 2024; Wang W. et al., 2024; Zhang et al., 2024). Against this backdrop, the Chinese Ministry of Education issued a policy to eliminate “low-quality courses” and cultivate “high-quality curricula,” explicitly urging undergraduates to enhance classroom engagement and learning concentration [Chinese Ministry of Education (CME), 2018]. Sustained concentration in learning not only awakens innovative potential but also fulfills the mission of advancing human civilization and shaping the future of society (Emara et al., 2025; Hu, 2025; Miller and Unsworth, 2025).

Learning concentration, defined as the learners’ ability to maintain concentration with academic content through volitional effort, while resisting distractions, is a critical educational construct that directly influences academic performance and productivity over time (Burke and Ray, 2008). Empirical studies consistently have demonstrated positive correlations between learning concentration and students’ cognitive abilities, academic achievement, and learning outcomes (Gallen et al., 2023; Liu, 2023; Safari and Yaghoobi, 2024). By encouraging active participation in learning activities, learning concentration serves as the foundation for stimulating critical thinking, fostering in-depth learning, and ensuring both pedagogical efficacy and educational quality. Furthermore, the post-pandemic era has seen online learning emerge as a dominant yet challenging mode of education, with digital distractions and fragmented attention posing significant threats to learning concentration (Emara et al., 2025; Guo, 2021; Lemay et al., 2021). As AI and digital tools become ubiquitous, students increasingly struggle to filter out overwhelming information and refocus on meaningful learning. Therefore, in an age of digitization and AI-driven education, unraveling the mechanisms of learning concentration and mitigating academic distractions hold urgent practical significance.

On September 26, 2024, China’s State Council Information Office emphasized the importance of children’s and adolescents’ healthy growth, well-being and holistic development at a press conference on high-quality education reforms, announcing an extension of class breaks from 10 to 15 min. This policy shift reflects a student-centered approach to education, prioritizing comprehensive growth over mere academic output, considering the most concerned issues for every family and the simplest wishes of parents. Also, robust empirical evidences highlight the benefits of micro-breaks, which reduce fatigue, promote relaxation, rejuvenate energy, and enhance focus, efficiency, well-being, and work performance (Albulescu et al., 2022; Conlin et al., 2021; Cropley et al., 2023; Lyubykh and Gulseren, 2023; Nomura et al., 2024; Schabram and Barnes, 2025). Furthermore, Barnes et al. (2007) found that psychological detachment is beneficial for employees to restore cognitive and emotional resources, improve interpersonal interaction quality, and mediate the relationship between micro-breaks and well-being (Garland et al., 2015), as well as the relationship between micro-breaks and attention (Bennett, 2015). However, the mechanisms through which micro-breaks influence concentration remain underexplored, akin to a “black box” requiring further and systematic investigation (Biwer et al., 2023; Dianita et al., 2024; Kitayama et al., 2022). Previously, there was not much research attention paid to micro-breaks in the field of study (Larson, 2006), and the same problems are even more severe for micro-breaks in the field of study. In today’s fast-paced social environment, maintaining sustained concentration and productivity is absolutely challenging (Mijović et al., 2015; Reed, 2025). While, it is even more urgent and important to explore how micro-breaks between study sessions (MBSS) can positively affect students’ learning concentration from a psychological perspective, such as the factors influencing study detachment and positive emotions. Therefore, building on these policy initiatives, empirical findings, and practical challenges, this study empirically examines how MBSS affect learning concentration, aiming to decode the “black box” of their mechanisms and advance both theoretical understanding and practical applications.

### MBSS and learning concentration

Micro-breaks, refer to short breaks taken during the completion of a task, typically defined as interruptions shorter than 10 min (Henning et al., 1989). Previous research has predominantly focused on the workplace micro-breaks, also termed “Micro-Breaks Between Works,” which are defined as “voluntary, non-institutionalized activities not mandated by organizational policies that temporarily interrupt continuous work.” These breaks are recognized as cost-effective, highly feasible strategies for employees to maintain or improve occupational health during the continuous work (Kim et al., 2017, 2018; Nie et al., 2020). That is, micro-breaks in the workplace emphasize acceptable and non-intrusive recovery behaviors in organizational or work contexts, and have more positive psychological value. Psychological detachment more often refers to a sustained attitude state, manifested as widespread indifference and low commitment, which differs significantly in performance and function from micro-breaks during work hours (Allam, 2017). If one continues to think about work, difficult problems, check anxious work emails, or engage in tasks that require focused attention during rest, it is an ineffective rest, let alone a micro-breaks during work hours. Cognitive recovery refers to the process of restoring and improving an individual’s cognitive functions (such as attention, memory, decision-making ability, etc.) that have declined due to fatigue, stress, or overuse through specific conditions or activities. It is an internal state change process, and at any time, the brain can be repaired from fatigue and regain the ability to process information efficiently through rest or environmental support (Kaplan, 1995; Stevenson et al., 2019). Micro-breaks is an external intervention or behavior in the workplace that can be taken during the workday, promoting cognitive recovery (Kim et al., 2017, 2018; Nie et al., 2023). Currently, while existing studies primarily examine workplace stress in professional settings, similar weekly stress cycles occur among students, students can mitigate severe long-term stress effects through micro-breaks between study sessions (MBSS), with recovery benefits comparable to those observed in workplace contexts (Larson, 2006). This study suggests that MBSS, specifically, refers to brief interruptions voluntarily initiated by students during self-directed and continuous learning periods (such as during self-study and homework completion, and the interruption time is usually around 10 min, controlled within 5–15 min). Academic settings demand extensive periods of learning, including reading course materials, writing lab reports and projects, taking exams, and engaging in other cognitively demanding activities, prolonged mental fatigue during these tasks may diminish the effectiveness of students’ efforts, ultimately leading to declines in academic performance (Kaplan, 1995; Tennessen and Cimprich, 1995). However, evidence from educational research suggested that MBSS allow the brain to enter an “offline” state, stabilizing previously acquired knowledge while enhancing learning concentration for subsequent tasks (Brokaw et al., 2016). Study on training programs further revealed that MBSS—particularly those involving brief eye closures—improve memory consolidation and retention of content compared to other cognitive activities (Dewar et al., 2007). Research involving college students also indicated that MBSS positively influenced task performance, especially in complex experimental settings, by boosting concentration (Chen Q., 2022). The mechanisms underlying micro-breaks’ capacity to enhance work concentration, vitality, well-being, and performance can be explained through Conservation of Resources (COR) Theory (Steed et al., 2021). COR posits that individuals instinctively acquire, protect, and nuture resources to sustain or foster new ones (Hobfoll, 1989). During intensive learning, of course, prolonged mental exertion depletes these resources. To maintain engagement, individuals must conserve existing resources or generate new ones. Also, micro-breaks replenish depleted resources through brief recovery periods, thereby energizing subsequent learning activities (Butze et al., 2021). Similarly, short breaks during academic sessions prevent energy depletion caused by sustained focus, ensuring adequate resources for optimal concentration in subsequent tasks. Based on this analysis, the present study proposes the following hypothesis:

*Hypothesis 1:* MBSS is positively related to student’s learning concentration.

### Mediating role of study detachment

Recovery Experience (RE), serves as a theoretical framework to explain the positive effects of MBSS, which refers to the degree to which non-work activities facilitate the restoration of energy resources, particularly through the emotional and psychological detachment from work-related demands during leisure time (Bosch et al., 2018; Trougakos et al., 2008). Among these experiences, psychological detachment is an important type of RE, which emphasizes the intentional disengagement from work-related thoughts and physical tasks during non-work periods. This process marks a psychological “switch” from a state of stress to recovery, enabling individuals to replenish depleted resources (Sonnentag, 2012). Empirical studies have linked psychological detachment to improved work focus and well-being. For instance, research on early childhood teachers highlights how high teaching stress in educational contexts may exacerbate risks to work engagement and health due to insufficient detachment (Lanaj et al., 2014). Extending this concept to student populations, researchers have adapted the framework of psychological detachment to propose study detachment, defined as a state where students exhibit behavioral and psychological disengagement from academic tasks during non-study periods, coupled with positive emotional experiences (Zhu, 2021). In academic settings, MBSS allow students to temporarily step away from learning tasks, achieving psychological detachment from academic stressors. This detachment facilitates the restoration of energy resources, which subsequently enhance subsequent learning engagement and concentration. Additionally, the Effort-Recovery Model (ERM) further supports this mechanism, positing that individuals must exert effort to meet task demands, but once such demands cease, physiological and psychological recovery processes reverse the strain and restore equilibrium (Fritz and Sonnentag, 2005; Zhu et al., 2019). Empirical evidence confirms that study detachment significantly alleviates learning-related stress and improves learning concentration among middle school students (Zhu, 2021). Thus, when students disengage from cognitively demanding tasks during micro-breaks, the cessation of effort expenditure triggers recovery processes, replenishing resources critical for sustaining learning concentration in subsequent learning activities. Building on this theoretical and empirical foundation, the present study proposes the following hypothesis:

*Hypothesis 2:* Study detachment mediates the relationship between MBSS and students’ learning concentration.

### Mediating role of positive emotions

Positive emotions, defined as pleasant affective states arising from the fulfillment of personal needs, exert multifaceted influences on physical health, work performance, and social functioning (Yang et al., 2016). Research indicated that employees in positive emotional states demonstrate heightened concentration and superior job performance (Tsai et al., 2012). Similarly, studies involving students reveal a robust positive correlation between positive emotions and learning engagement, with learning concentration as a core dimension of engagement, which exhibiting even stronger associations with positive affect (Yan et al., 2024). For instance, research on proactive personality traits in college students demonstrated that positive emotions mediated the relationship between proactive personality and learning engagement (Bao et al., 2022). Also, MBSS correlate positively with positive emotions (Biwer et al., 2023; Butze et al., 2021). Grounded in Affective Events Theory (AET), workplace or academic events can act as proximal triggers for short-term emotional responses. MBSS, conceptualized as affective events during learning periods, may elicit positive emotions when students engage in relaxing activities (e.g., casual conversations during breaks or browsing favorite sports content), thereby fostering satisfaction and enjoyment (Walter and Bruch, 2009). Furthermore, ERM posits that psychological recovery occurs when individuals disengage from work- or learning-related demands, leading to improved emotional states (Fritz and Sonnentag, 2005). When a person is away from work/study demands, their psychological state will return to the level before the demands, and their emotional condition will improve. And during the MBSS, participation in preferred leisure activities not only restores mental equilibrium but may also amplify positive affect. Empirical evidence consistently underscored the role of micro-breaks in enhancing positive emotions (Dianita et al., 2024; Kim et al., 2022; Kitayama et al., 2022; Lyubykh and Gulseren, 2023; Schabram and Barnes, 2025; Scholz et al., 2018). The above analysis suggests that positive emotions induced by MBSS serve as a critical mediator in the relationship between MBSS and learning concentration. The present study proposes the following hypothesis:

*Hypothesis 3:* Positive emotions mediates the relationship between MBSS and students’ learning concentration.

### Chain mediating pathway

Study detachment and positive emotions may independently or sequentially mediate the relationship between MBSS and learning concentration. The effort-recovery model suggests that micro-breaks activities are crucial for individual resource recovery, as they facilitate the recovery of functional systems such as emotions and cognition from the accumulated load of continuous work (Bennett et al., 2016; Meijman and Mulder, 1998). Based on the effort-recovery model, employees need to take micro-breaks during long hours of work to restore their normal physical function and prevent physical or psychological discomfort caused by overloaded work requirements (McLean et al., 2001; Nie et al., 2023). Micro-breaks can help employees temporarily achieve psychological detachment from work, thereby assisting them in restoring their own resources such as emotions and attention (Bennett, 2015; Chaikumarn et al., 2018; Dababneh et al., 2001; Hunter and Wu, 2016). To the same as, the effort-recovery model is applicable to students and MBSS (Bennett et al., 2020; Gabriel et al., 2019), which can help students temporarily detach from learning, thereby helping them recover their emotions and concentration. Grounded in the AET, workplace events trigger individual experiences, which evoke emotional responses that further shape attitudes and behaviors. One pathway through which emotional responses influence behavior is by directly affecting behavioral outcomes (Wang S. et al., 2024; Wang W. et al., 2024; Weiss and Cropanzano, 1996). As a positive emotional event, MBSS first induces study detachment (a recovery experience), and the subsequent positive emotions generated by this detachment serve as a critical mechanism for enhancing learning concentration. Existing research demonstrated that psychological detachment alleviates workload strain, while positive emotions replenish employee vigor, collectively providing the resource conditions necessary for sustained work concentration (Tsai et al., 2012). Similarly, students who engage in MBSS gain recovery through study detachment and positive emotions, which restore their cognitive and emotional resources, thereby supporting subsequent learning concentration. Building on these findings, the present study proposes the following hypothesis:

*Hypothesis 4:* Study detachment and positive emotions play the chain mediating role between MBSS and students’ learning concentration.

**Figure 1:**
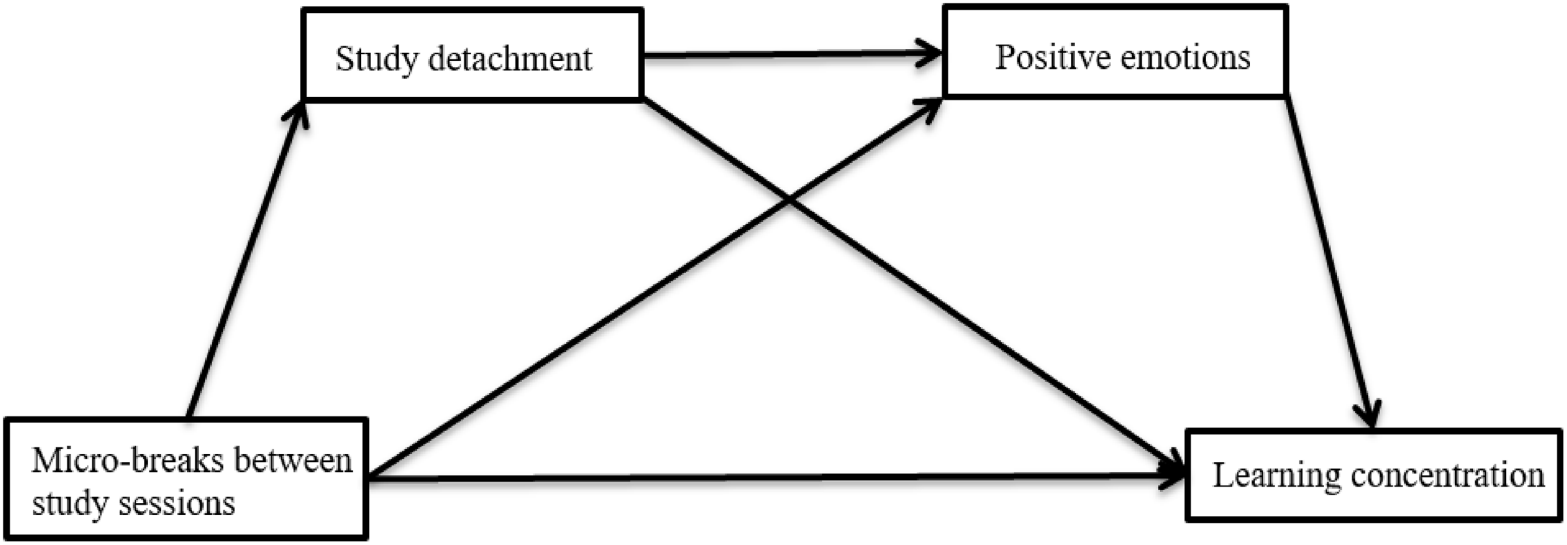
Research proposed model.

This study employed a sample of college students to investigate MBSS as the independent variable, learning concentration as the dependent variable, and study detachment and positive emotions as mediators. The analysis examined both the unique and sequential mediation effects of study detachment and positive emotions in the association between MBSS and learning concentration. By elucidating the mechanisms underlying learning concentration through the lens of MBSS, this research aims to decode the “black box” of concentration regulation, offering actionable strategies to help students balance academic concentration with leisure activities. The hypothesized model is illustrated in Figure 1.

## Methods

### Procedures

This method was reviewed and approved by the morality and ethics committee of the Public Course Teaching Department of Shandong University of Science and Technology, and the participants provided orally informed consent to this study. Firstly, six psychology graduate students translated the original scales into Chinese and modified the work scenario into a study scenario. After being translated back by English professionals to ensure consistency in the meaning of the scales between Chinese and English, a psychology expert discussed and finalized the revised version with the researchers. Secondly, during the break between classes, students fill out the Chinese version of the questionnaire, which will be collected on the spot 10 min later. Any incomplete questionnaire will no longer be collected. After all questionnaires are collected, they will be discarded and questionnaires with missing basic information, excessive blank spaces, and overly obvious reaction tendencies will be removed. Finally, valid questionnaires will be obtained for statistical analysis.

### Participants

This study employed a convenience sampling method to recruit college students from three normal universities in Shandong Province. These students are all full-time undergraduate students who mainly participate in traditional face-to-face teaching activities. Their average course load is about 20–25 credit hours per week. Their common micro-breaks behaviors include using mobile phones (such as browsing social media, watching short videos), talking to classmates, taking walks, closing their eyes to rest, and eating snacks. The typical micro-breaks frequency is once every 1–2 h of learning, with a single micro-break time of 5–15 min. A total of 600 questionnaires were distributed, with 578 valid responses returned, yielding a valid questionnaire rate of 96.3%. The demographic characteristics of the participants are summarized as follows: in terms of gender, there were 256 male students (44.3%) and 322 female students (55.7%); the age of the participants ranged from 17 to 23 years old, with a mean age of 18.72 years (*M* = 18.72, *SD* = 1.03); regarding the grade level, there were 194 first-year students (33.6%), 206 s-year students (35.6%), and 178 third-year students (30.8%); in terms of whether they were student leaders, there were 180 student leader (31.1%) and 398 non-student leader (68.9%); regarding whether they were only children, there were 206 only-children (35.6%) and 372 non-only children (64.4%).

### Measurements

#### MBSS scale

The MBSS Scale was adapted from the Micro-breaks Scale developed by the workplace (Kim et al., 2017). While, the MBSS referred to in this study specifically refers to the voluntary and brief interruptions initiated by students during self-directed and continuous learning periods, such as self-study and homework completion. The original 9-item scale, modified by replacing work-related terms with learning contexts, includes items such as “During long periods of study, taking a short break and doing things unrelated to learning.” Responses are recorded on a 5-point Likert scale (1 = *Strongly disagree*, 5 = *Strongly agree*), with higher scores indicating longer micro-breaks between study sessions. Cronbach’s *α* for this scale was 0.81. Confirmatory factor analysis (CFA) revealed good reliability and structural validity, with χ^2^/df = 3.03, CFI = 0.92, GFI = 0.91, IFI = 0.87, and RMSEA = 0.078.

#### Study detachment scale

The Study Detachment Scale was adapted from the Recovery Experience Scale developed by the workplace (Sonnentag and Fritz, 2007), focusing on the psychological detachment dimension. The original 4-item scale, modified for academic contexts (e.g., “After studying, I do not think about academic tasks at all”), uses a 5-point Likert scale (1 = *Strongly disagree*, 5 = *Strongly agree*), with higher scores reflecting greater psychological detachment. Cronbach’s α for this scale was 0.86. CFA results indicated good fit: χ^2^/df = 2.89, CFI = 0.98, GFI = 0.98, IFI = 0.97, and RMSEA = 0.051.

#### Positive emotions questionnaire

The Positive Affect subscale of the Positive and Negative Affect Schedule (PANAS) (Watson et al., 1988), was used to measure positive emotions. This 10-item scale employs a 5-point Likert scale (1 = *Very slightly or not at all*, 5 = *Extremely*), with higher scores indicating stronger positive emotions. Sample items include “Excited” and “Enthusiastic.” Cronbach’s α for this scale was 0.89. CFA demonstrated adequate validity: χ^2^/df = 3.02, CFI = 0.93, GFI = 0.91, IFI = 0.93, and RMSEA = 0.085.

#### Learning concentration scale

The Learning Concentration Scale was adapted from the Learning Engagement Scale (Fang et al., 2008). The 3-item scale, rated on a 5-point Likert scale (1 = *Strongly disagree*, 5 = *Strongly agree*), assesses focused engagement in learning (e.g., “I am fully immersed in my learning”). Cronbach’s α for this scale was 0.83. CFA results confirmed good reliability and structure: χ^2^/df = 2.97, CFI = 0.89, GFI = 0.90, IFI = 0.89, and RMSEA = 0.075.

### Data analysis

Data were analyzed using SPSS 22.0 for descriptive statistics, correlation analysis, and mediation effect testing via the PROCESS macro (Version 4.2). Bootstrapping with 5,000 resamples and a 95% confidence interval was applied to examine indirect effects.

## Results

### Common method bias test

Harman’s single-factor test was conducted to assess common method bias (Podsakoff et al., 2003). The results revealed that the KMO value was 0.84 (*p* < 0.001), indicating that the scales were suitable for factor analysis. And the results revealed 8 factors with eigenvalues greater than 1, and the first factor explained 23.99% of the variance, which was below the critical threshold of 40%. This indicates that common method bias was not a significant concern (Fournier et al., 2021).

### Confirmatory factor analysis

In order to test the discriminant validity among the four variables involved, this study conducted confirmatory factor analysis (CFA) to examine the construct validity between the four constructs of MBSS, study detachment (SD), positive emotions (PE), and learning concentration (LC), and AMOS 24.0 was used to compare the differences in fit between the four-factor model (Model 4), three-factor model (Model 3), two-factor model (Model 2), and one-factor model (Model 1) (Wang et al., 2005). The results of confirmatory factor analysis are shown in Table 1. The fitting indicators of the four-factor model have reached acceptable standards and are significantly better than other candidate models, indicating that these four variables have different constructs and good construct validity (Zeng et al., 2018; Zeng and Yan, 2013; Hu and Wang, 2014).

**Table 1:** Confirmatory factor analysis to assess construct validity.

Model	Factor loaded	χ^2^/df	df	CFI	TLI	RMSEA	SRMR
Model 4	Four factors: MBSS, SD, PE, LC	733.78	293	0.95	0.93	0.05	0.06
Model 3	Three factors: MBSS, SD, PE + LC	1075.66	296	0.83	0.81	0.09	0.12
Model 2	Two factors: MBSS+SD, PE + LC	1227.99	298	0.68	0.65	0.10	0.14
Model 1	One factor: MBSS+SD + PE + LC	1575.94	299	0.56	0.52	0.12	0.17

SD = Study detachment, PE = Positive emotions, LC = Learning concentration, + = the combination of two factors into one factor.

While, to further prove that there is no important problem with common method bias, this study once again used confirmatory factor analysis with labeled variables for testing. Construct a nested model with “sleep quality (SQ)” as the marker variable, which includes five core latent factors: “MBSS, SD, PE, LC, and SQ,” as well as one common method factor (Podsakoff et al., 2003). Comparing the baseline model without method factors with the labeled variable model with method factors (Conway and Lance, 2010), it was found that the fitting degree of the labeled variable model was significantly improved (ΔCFI = 0.023, ΔRMSEA = − 0.008). Furthermore, according to the convention for comparing nested models (Cheung and Rensvold, 2002), a ΔCFI ≥ 0.010 is generally regarded as the threshold for a substantial improvement in model fit. In this study, the ΔCFI of 0.023 well exceeds this threshold, indicating that the inclusion of the method factor significantly enhanced the model’s explanatory power for the data. Meanwhile, the ΔRMSEA of −0.008 showed the observed decrease in ΔRMSEA aligns with the fundamental principle that ‘a lower value indicates better model fit’ (Steiger, 2007). This combined pattern of results is consistent with the typical application of the CFA marker technique for controlling common method bias (Williams et al., 2010). Specifically, the method factor effectively captured a portion of the systematic variance attributable to the method (Podsakoff et al., 2003), thereby improving the fit of the measurement model without distorting the structural relationships among the core constructs (Richardson et al., 2009). This suggests that the influence of common method bias on testing the core hypotheses in this study was within an acceptable range (Chen, 2007; Widaman, 1985).

### Descriptive statistics and correlations analysis

The means, standard deviations, and correlation matrix of key variables are presented in the Table 2. The correlation analysis showed no significant gender or grade differences in MBSS or learning concentration. However, student leader was significantly positively related to learning concentration (r = 0.25, *p* < 0.01), while being an only child significantly positively correlated with study detachment (r = 0.15, *p* < 0.01) and learning concentration (r = 0.10, *p* < 0.05). All main variables that MBSS, study detachment, positive emotions, and learning concentration, exhibiting significant positive correlations (*p* < 0.01).

**Table 2:** Descriptive statistical results and correlation coefficients.

Variables	*M*	*SD*	1	2	3	4	5	6	7	8
1 G	-	-	1							
2 GL	-	-	0.01	1						
3 SL	-	-	0.00	0.04	1					
4 OC	-	-	0.20^**^	0.07	−0.06	1				
5 MBSS	3.31	0.56	−0.06	0.10	0.09	0.09	1			
6 SD	3.25	0.75	0.10	0.07	−0.03	0.15^**^	0.19^**^	1		
7 PE	3.11	0.78	0.10	−0.01	0.11	−0.01	0.22^**^	0.23^**^	1	
8 LC	3.36	0.79	0.04	0.07	0.25^**^	0.10^*^	0.22^**^	0.26^**^	0.33^**^	1

^**^p < 0.01, ^*^p < 0.05; G = Gender, GL = Grade level, SL = Student leader, OC = Only-child, SD = Study detachment, PE = Positive emotions, LC = Learning concentration.

### Chain mediating effect test

After standardizing the variables, MBSS was treated as the independent variable, learning concentration as the dependent variable, and study detachment and positive emotions as sequential mediators. Student leader and only-child were included as control variables. Bootstrapping with 5,000 resamples (95% confidence interval) was applied using PROCESS Macro to examine indirect effects. The statistical results are shown in Table 3.

**Table 3:** Results of regression analysis.

Predictors	LC			SD			PE			LC		
	*β*	*SE*	*t*	*β*	*SE*	*t*	*β*	*SE*	*t*	*β*	*SE*	*t*
SL	0.161	0.099	1.637	−0.054	0.094	−0.571	0.396	0.094	4.216^***^	0.071	0.096	0.743
OC	0.116	0.095	1.218	0.214	0.091	2.359^*^	−0.058	0.092	−0.628	0.080	0.091	0.882
MBSS	0.292	0.081	3.593^**^	0.240	0.078	3.096^**^	0.227	0.079	2.882^**^	0.177	0.079	2.226^*^
SD							0.207	0.059	3.500^**^	0.183	0.060	3.044^**^
PE										0.259	0.059	4.398^***^
R^2^	0.062			0.056			0.137			0.167		
F	6.326^**^			5.604^**^			11.274^***^			11.346^***^		

^***^p < 0.001, ^**^p < 0.01, ^*^p < 0.05; G = Gender, GL = Grade level, SL = Student leaders, OC = Only-child, SD = Study detachment, PE = Positive emotions, LC = Learning concentration.

**Figure 2:**
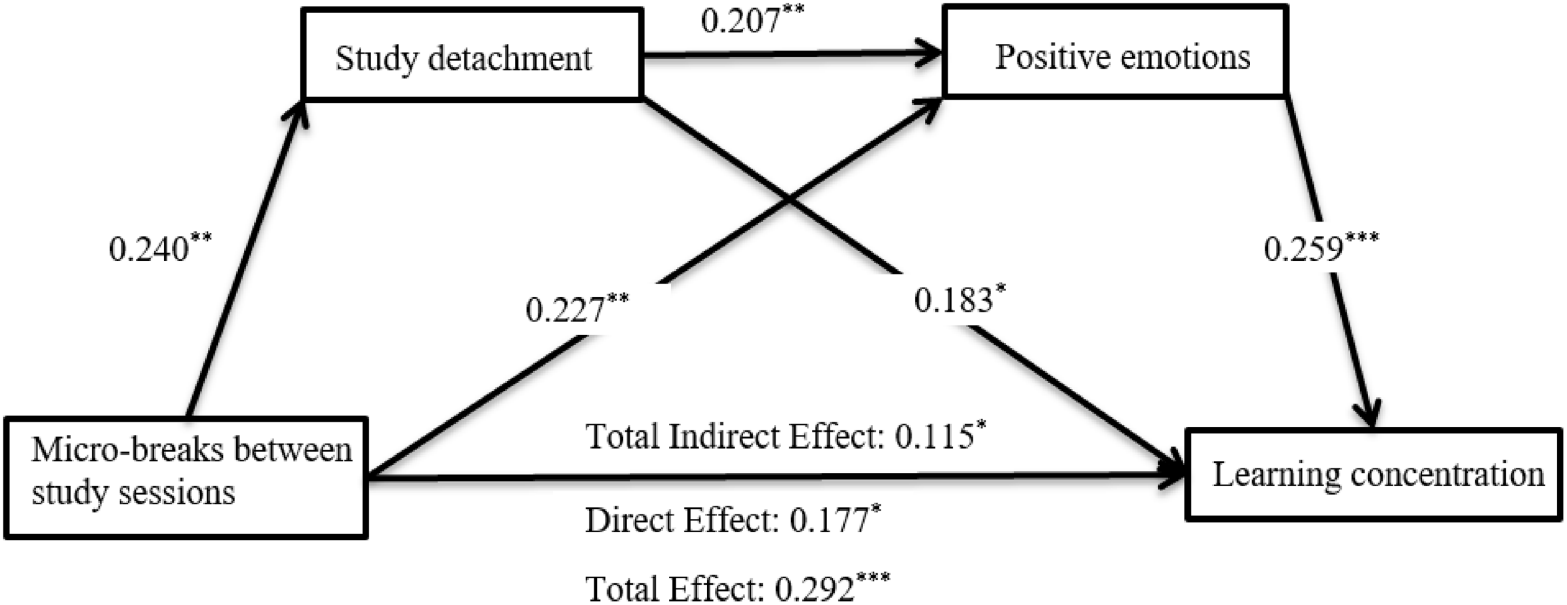
Path analysis of chain mediating effect test.

As shown in the regression analysis (Table 3), after controlling for student leader and only-child, MBSS was significantly positively related to learning concentration (*β* = 0.292, *t* = 3.593, *p* < 0.01). When study detachment and positive emotions were included in the regression model, MBSS significantly predicted study detachment (β = 0.240, *t* = 3.096, *p* < 0.01) and positive emotions (β = 0.227, *t* = 2.882, *p* < 0.01). Study detachment was significantly positively associated with positive emotions (β = 0.207, *t* = 3.500, *p* < 0.01) and learning concentration (β = 0.183, *t* = 3.044, *p* < 0.01). Positive emotions was also significantly positively correlated with learning concentration (β = 0.259, *t* = 4.398, *p* < 0.001). Notably, the direct effect of MBSS on learning concentration remained significant (β = 0.177, *t* = 2.226, *p* < 0.05). The mediation effects are summarized in Table 4 and Figure 2. The total effect of MBSS on learning concentration was 0.292 (95% CI [0.132, 0.452], *p* < 0.001), indicating a statistically significant total effect. The direct effect of MBSS on learning concentration was 0.177 (95% CI [0.020, 0.333], *p* < 0.05), confirming its direct significance. The total indirect effect through study detachment and positive emotions was 0.115 (95% CI [0.054, 0.192], *p* < 0.001), demonstrating significant mediation.

**Table 4:** Analysis of the mediating effect of LC and PE.

Indirect effects	Effect value	Boot SE	95% Confidence interval	
			Lower limit	Upper limit
Total effect	0.292	0.081	0.132	0.452
Direct effect	0.177	0.079	0.020	0.333
Total indirect effect	0.115	0.036	0.054	0.192
Indirect effect 1	0.044	0.024	0.008	0.105
Indirect effect 2	0.013	0.007	0.004	0.031
Indirect effect 3	0.059	0.026	0.018	0.118

## Discussion

### Corelationship of MBSS and learning concentration

This study demonstrated that MBSS was significantly positively related touniversity students’ learning concentration, validating the hypothesis 1. This research result is consistent with the COR, because that, university students’ learning is a resource-depleting process, particularly, when sustained concentration is required, over time, prolonging mental exertion drains cognitive and emotional resources, whereas MBSS disrupt this cycle by providing opportunities for resource replenishment, thereby supporting sustained concentration in subsequent learning tasks (Butze et al., 2021). Such as temporarily quitting the learning tasks, which enable students to recover cognitive and emotional resources, fostering a state of mental flexibility and emotional readiness that enhances concentration in future learning tasks, just as being consistent with the previous research findings (Brokaw et al., 2016; Butze et al., 2021; Trougakos et al., 2008). Also, gazing out the window for the scenery through simple MBSS, which can restore the students’ learning concentration (Tennessen and Cimprich, 1995). At the same time, this can also support the research result of this article, based on the perspective that happiness is productivity, that is, MBSS can make individuals more pleasure and joyful in learning. As time goes by, students will posit the learning as a source of joy, thereby gradually cultivating their learning concentration and intrinsic motivation (Chong et al., 2020). What is commendable is that, the results of this study draw inspiration from workplace micro-breaks research to the academic domain, confirming the cross-contextual applicability of MBSS as an energy-management strategy for learners, and verifying consistency with relevant researches on student groups and other work groups (Larson, 2006; Zhu et al., 2019). As students, this further proves that, MBSS, such as engaging in appropriate leisure and relaxation activities in addition to intense learning can help improve their subsequent learning tasks.

### Mediating role of study detachment

The results of this study identified that, there is a separate and significant mediating effect of study detachment between MBSS and learning concentration, supporting the hypothesis 2. Recovery theory can explain the mediating mechanism of the effect of MBSS on interpretability, micro-breaks activities can help employees temporarily achieve psychological detachment from work, thereby helping them restore positive emotions and attention (Bennett et al., 2016; Meijman and Mulder, 1998), which indirectly proves the conclusion of this study. Additionally, MBSS helps individuals achieve study detachment, obtain relaxation experiences, thus, and improve physical and mental health and work status (Garland et al., 2015), confirming the research result of this study. During the heavy learning tasks, MBSS, activities like stretching, strolling outside the classroom, or exchanging interesting topics with other students, which can achieve physical and mental relaxation, providing the best state for the next class tasks and helping to restore more concentration during the learning period. This is consistent with previous research findings (Zhu, 2021).

Psychological detachment can play a mediating role between micro-breaks and positivity (Chong et al., 2020), further validating the result of this study. Similarly, during the learning period, MBSS can free students from academic pressure, achieve study detachment, enhance relaxation experience, and reduce or even eliminate cognitive tension, thereby improving students’ subsequent learning vitality and concentration (Zhu, 2021). Under the condition that students close their eyes and rest for a few minutes after learning or testing, they can perform more concentration and better in subsequent learning tasks. The important mechanism behind this is that students’ brains temporarily leave the learning context, which is the mediating effect of study detachment (Liu, 2024). Collectively, whether it is a micro-breaks in the work environment or MBSS, temporarily relieving oneself from work/study is an important mechanism source for producing excellent performance afterwards, also inspiring us to engage in MBSS activities after long-term learning, which can indeed alleviate learning fatigue and improve learning performance.

### Mediating role of positive emotions

Also, positive emotions are underscored as a unique mediator between MBSS and learning concentration, corroborating the hypothesis 3. Rather than directly generating positivity, MBSS create opportunities for students to engage in preferred restorative activities (e.g., watching humorous videos, meditating, or chatting casually), which inherently reduce resource depletion and amplify positive affect (Trougakos and Hideg, 2009). During the worktime, employees utilize these micro-breaks to better prevent the depletion of resources required by job demands, and engage in recreational activities that, being characterized by low effort and are preferred choices by individuals, in order to facilitate effective recovery (Lanaj et al., 2014). These type of activities themselves do not require the consumption of the individual’s existing resources, but by investing in activities that are preferred and enjoyed by the individual, resources can be accumulated, which in turn brings significant benefits for better work and learning, as well as building job satisfaction (Kim et al., 2022), indirectly proving the conclusion of this study. Engaging in activities one enjoys during work micro-breaks can bring joy to both body and mind, leading to more positive emotions and a sense of happiness. These conclusions have been supported by numerous studies (Fritz and Sonnentag, 2005; Trougakos et al., 2008; Zhao and Zhao, 2023). Meanwhile, such activities align with the “happy is productivity” principle, where low-effort, enjoyable pursuits replenish resources and bolster resilience against academic stress (Chong et al., 2020). Experimental evidence further found that, positive emotions like, when people feel happy, the levels of a chemical called cortisol in the brain decrease, the immune system improves, and they feel more empowered and courageous to adjust to difficult tasks, maintaining persistence and concentration on difficult tasks (Lanaj et al., 2014). On the other hand, in the context of campus learning, during the learning period, MBSS, such as the students engage in low effort and their preferred entertainment activities during micro-breaks in their studies. For example, some students enjoy watching relaxing short videos, and the humorous content in these videos instantly ignites their enthusiasm; some students like to chat with other classmates, and the content of the chat is full of humor; some students like to meditate and imagine themselves in very relaxed situations. These micro-breaks activities will prevent resource depletion caused by the continuous learning content, and thus provide resources for future learning engagement, which is beneficial for maintaining learning concentration.

### Chain mediating effect of study detachment and positive emotions

The most striking finding is the chain mediating effect of study detachment and positive emotions in the relationship between MBSS and learning concentration, confirming the hypothesis 4. The learning tasks of modern university students are quite heavy, with a very tight schedule of courses every day and a large amount of homework, and participation in other student activities, which leads to a lot of pressure on students (Pang et al., 2021). Meanwhile, with the increasing demand for undergraduate teaching, the evaluation and assessment of courses have placed more emphasis on process evaluation and diversity of evaluation. Therefore, in addition to delivering excellent courses, students also need to complete various evaluation stages, facing with increasing academic pressure, who can take MBSS to temporarily free from the continuous consumption of courses, immerse themselves in non-learning activities they enjoy, build positive emotions and feelings of happiness, and provide a source of energy for future course learning, which is more conducive to learning concentration and effectiveness. Wherein, some studies have also confirmed this conclusion. Among them, the results of meta-analysis indicated that individuals with positive emotions and a focus on getting rid of tasks are more likely to engage and concentrate on work (Steed et al., 2021). The research results of the experience sampling method verified the mechanism of the effect of micro rest during work hours on work engagement among employees in private enterprises, and verified the chain mediated role of recovery experience and positive emotions in it (Chen X., 2022). In a memory experiment task, the influence of micro-breaks and without task encoding on micro-breaks results was explored, and the research results of the student population showed that micro-breaks after the encoding task had the same positive effect as micro-breaks after the test (Poskanzer et al., 2021). The results of this study are consistent with the above research conclusions. As a consequence, this study took advantage of the university students as the group and verified the important role of MBSS in the student population through empirical investigation, and further proved the substance of balancing work and rest for students during the learning period.

### Suggested strategy

#### Guiding college students to pay attention to MBSS

Given the significant positive correlation between MBSS and learning concentration, college students should not only study hard, but also fully utilize micro-breaks to engage in recreational activities. And every college student should choose low-energy and more interesting non learning activities based on their own interests. Because these activities are characterized by low-intensity or relaxed activities, they are associated with lower physical and mental fatigue and higher positive emotions (Fritz et al., 2011; Sianoja et al., 2018). Students who are accustomed to engaging in interpersonal interaction activities with others during micro-breaks can achieve social support by communicating with familiar classmates; Some students are accustomed to achieving spiritual detachment from learning tasks through recreational cognitive activities such as leisure reading or online entertainment; Other students achieve physical and mental relaxation by stretching their bodies or practicing mindfulness meditation during micro-breaks. At the same time, it is more important to solidify suitable MBSS activities as a long-term habit, and to better utilize their cumulative effects through continuous use.

#### Reasonably arrange rest and study time

The break between classes is formulated according to the education department, which is very important for learning efficiency and cannot be ignored in school education (Liu, 2024). Therefore, from the perspective of break time, as a teacher, it is important to ensure that the regular ten to fifteen minute break does not drag the class, and encourage students to engage in their preferred leisure activities during break time, so that students fully realize that leisure breaks are also an essential part of their growth and development. In addition, as the management department of the school, while providing students with sufficient and superior learning environment, should strengthen the cultural atmosphere construction of reasonable arrangement of learning time and break time, actively guide teachers and students to change traditional concepts, and change the erroneous notion that only by filling time and constantly learning can learning improve learning effectiveness, and that leisure and entertainment in learning is a manifestation of lack of focus. Because taking breaks between classes can help students better allocate cognitive resources, improve learning efficiency, and cultivate interpersonal communication skills (Ramstetter et al., 2010).

Reasonable arrangement of classroom teaching based on the cognitive characteristics of college students.

Educators need to understand the psychological characteristics of college students from the perspectives of psychology and education, and combine them with the characteristics of learning activities to analyze the interests and concerns of students in learning, and improve the teaching mechanism from both teaching and learning aspects (Liu, 2022). As a full-time teacher of mental health courses, it is even more important to add leisure content to the curriculum design, such as incorporating positive psychology courses such as mindfulness and optimism into physical and mental health education, to help students master some basic mindfulness theoretical content and practical skills, such as mindfulness breathing, body scanning, and raisin exercises (Huong and Vu, 2023). By mastering these concepts and practices, can better improve the leisure effect during leisure time. Moreover, as full-time teachers of professional courses, should put effort into curriculum design, provide challenging and hierarchical learning tasks, use interactive and modern teaching tools and methods for teaching, timely recognize students’ progress and contributions, and enhance their sense of value and competence. In this way, improving the quality of MBSS through mindfulness education, enhancing students’ positive emotions and sense of participation in learning through professional curriculum education, and improving their concentration and efficiency in learning and break time.

### Theoretical significance and practical values

#### Theoretical significance

This study expands the theoretical framework of MBS and learning concentration through multidimensional innovations, offering the following academic contributions:

First, theoretical transfer and situational innovation. While prior research predominantly focused on workplace “micro-breaks,” this study pioneered the application of COR and ERM to educational contexts, which validated the direct effects and mediating pathways of MBSS on learning concentration, thereby extending the theoretical boundaries of micro-breaks research and introducing novel perspectives to educational psychology.

Second, deepening mechanistic insights. By elucidating the dual-path mediation (study detachment and positive emotions) and chain mediating mechanisms, this research integrated cognitive detachment and emotional restoration into a dynamic coupled system. This constructed a progressive model—"behavioral intervention → cognitive relief → emotional activation → enhanced concentration,” transcending traditional single-path explanations.

Third, expanding mediation models. The study confirmed the presence of chain mediation (MBSS → study detachment → positive emotions → learning concentration), aligning with the “event-cognition-affect-behavior” transmission logic of AET, which provided systematic theoretical support for understanding the mechanisms underlying learning concentration. Additionally, empirical validation of recovery experiences and emotional resource accumulation in university students filled a critical gap in student-focused micro-breaks research, laying the foundation for cross-population comparisons.

#### Practical values

The findings offered actionable guidance for enhancing learning concentration and efficiency and optimizing educational management:

First, the individual-level behavioral guidance. Students can strategically schedule MBSS (e.g., casual chats during breaks, light physical exercise, or mindfulness meditation) to achieve cognitive detachment and emotional recovery, thereby boosting subsequent learning concentration. The study recommended prioritizing low-effort, high-interest activities (e.g., watching humorous videos or social interactions) to maximize recovery effects.

Second, the improving educational management strategies. Schools should move away from rote-learning models, ensure adequate break durations, and eliminate prolonged classes. Campus culture should promote study-rest balance, such as incorporating mindfulness exercises or leisure education into curricula to equip students with scientific recovery techniques.

Third, the scientific basis for policy-making. The results provided empirical support for the Ministry of Education’s policy on extending class micro-breaks, advocating for further promotion of flexible learning arrangements. Educators should prioritize students’ recovery needs. As well as, developing MBSS-based interventions (e.g., “fragmented mindfulness training”) and deploying digital tools to monitor concentration fluctuations could enable personalized learning management.

### Limitations and future directions

Despite its contributions, this study has limitations: (1) Limited sample representativeness. Data were collected from three normal universities in Shandong Province, with participants concentrated in specific age groups and majors. Non-full-time or vocational college students were excluded, limiting the generalizability of conclusions; and (2) Cross-sectional design constraints. While mediation models explored variable relationships, causality between MBSS and learning concentration cannot be strictly inferred. Future longitudinal or experimental studies (e.g., randomized controlled trials comparing micro-break strategies) are needed; and (3) The data source is single. The same method—all self-reported questionnaires, the same source (all self-reported by students themselves), and the same time point (all single cross-sectional surveys)—cannot completely avoid common method bias, let alone fully confirm the relationships between variables; and (4) Measurement limitations. Especially, the use of a short three-item scale for learning concentration and the treatment of micro-breaks as a single construct despite different break types; and (5) Self-report bias. Measurements relied on subjective student reports, potentially influenced by social desirability. Future work should integrate physiological indicators (e.g., heart rate variability, EEG) or behavioral observations (e.g., classroom focus duration logs) for enhanced objectivity.

To address these limitations, future studies could explore: (1) Diverse data. By introducing objective data such as teachers’ ratings of students’ attention, other students’ ratings of their attention, and objective performance indicators, avoiding errors or potential impacts caused by purely subjective data; and (2) Experience sampling method. Predictive and outcome variables can be measured at different time points, or specific changes can be tracked by measuring several time periods per day, in order to more accurately measure state changes and causal relationships; and (3) Refine the behavior of MBSS. Due to the widespread integration of AI into smartphones, MASS can be accurately defined as short-term breaks for smartphones, such as browsing social media, short videos, playing games, etc. This makes it more suitable for specific groups of people (who have shorter and more random breaks), such as young, stressed, and energetic delivery workers and other emerging employment groups in China. A too broad definition or behavior of MBSS may not accurately determine its location in these specific groups, and its research conclusions may also be open to debate; and (4) Enrich the measurement questionnaires. Try to use the complete version of the questionnaires for measuring each variable as much as possible, in order to more comprehensively measure the connotation of each variable, such as the six items for learning concentration, rather than the simplified version of the three items. Especially for micro-breaks, it is necessary to conduct a detailed comparison and reflect the smart-phone micro-breaks (such as watching short videos, playing mobile games), rather than just traditional micro-breaks (such as eating snacks, drinking water) or neutral activities (such as meditation, writing diaries). This has important practical significance and theoretical value for in-depth research and comparison of different types of micro-breaks; and (5) Multidimensional mechanisms. Incorporate additional mediators (e.g., cognitive flexibility, self-regulatory efficacy) or moderators (e.g., personality traits, learning environments) to build more comprehensive models. Or control for potential variables such as fatigue, hunger, and hyperactivity that may affect research conclusions; and (6) Cross-cultural comparisons. Investigate cultural differences in micro-break patterns between Chinese students (often facing intense academic pressure) and Western peers, examining how socio-cultural contexts moderate outcomes; and (7) Technology-enhanced applied research. Develop AI-driven intelligent micro-break prompting systems that recommend personalized rest intervals based on real-time student states (e.g., fatigue levels, emotional fluctuations), evaluating long-term impacts on learning efficiency; and (8) Policy tracking. Conduct multi-wave longitudinal studies or experimental researches to quantify changes in student focus and mental health before and after implementing China’s “extended class break” policy, providing dynamic evidence for policy refinement.

## Conclusion

The conclusion of this study is that, there is a significant positive correlation between MBSS and learning concentration, which is mediated by study detachment and positive emotions respectively, forming a chain mediating model. This study has important theoretical significance and practical value for revealing the positive impact and mechanism of MBSS on learning concentration, as well as for education policy makers and teachers to adopt relevant policies and methods.

## Data Availability

The raw data supporting the conclusions of this article will be made available by the authors, without undue reservation.
